# Timely Resolution of SARS-CoV-2-Related Multi-System Inflammatory Syndrome in Children

**DOI:** 10.3390/v15010094

**Published:** 2022-12-29

**Authors:** Daniel D. Reiff, Randy Q. Cron

**Affiliations:** 1 Division of Rheumatology, Department of Pediatrics, University of Alabama at Birmingham, Birmingham, AL 35233-1711, USA; 2 Children’s of Alabama, Division of Rheumatology, 1600 7th Ave. S., Birmingham, AL 35233-1711, USA

**Keywords:** multi-system inflammatory syndrome in children, COVID-19, SARS-CoV-2

## Abstract

*Background*: Multisystem inflammatory syndrome in children (MIS-C) is a severe, postinfectious manifestation of coronavirus disease 2019 (COVID-19) in the pediatric population. The disease is manifested by hyperinflammation and can result in cardiac dysfunction, coronary changes, and end-organ damage. Adequate timely treatment can prevent poor outcomes in the short term, but long-term data is lacking. *Methods*: A large single center MIS-C cohort was followed longitudinally after treatment with intravenous immunoglobulin (IVIG) ± glucocorticoids to determine the natural history of the disease and to describe improvement in laboratory markers and cardiac outcomes. Patient were stratified by disease severity and compared. *Results*: 137 patients were identified with demographics similar to previously described cohorts. Regardless of disease severity, when adequately treated, initial lab abnormalities rapidly improved by the 6–8 month follow-up period, with some resolved in as little as 1–2 weeks. Similarly, cardiac abnormalities improved quickly after treatment; all abnormalities resolved in this cohort by 1–2 months post-hospitalization. *Conclusions*: Although MIS-C is a serious sequela of COVID-19, when identified quickly and treated aggressively, laboratory abnormalities, coronary dilatation, and systolic dysfunction rapidly improve with minimal long-term morbidity or mortality.

## 1. Introduction

Since severe acute respiratory syndrome coronavirus 2 (SARS-CoV-2) and coronavirus disease 2019 (COVID-19) first appeared in late 2019, pediatric patients have been uniquely affected by the hyperinflammatory, postinfectious phenomenon known as multisystem inflammatory syndrome in children (MIS-C). With its high levels of inflammation, release of pro-inflammatory cytokines, and notable laboratory abnormalities leading to severe hypotensive shock, depressed cardiac function and coronary changes, and end-organ dysfunction, MIS-C has been an alarming disease process that has affected thousands of children across the United States and the world [1,2]. Fortunately, given its similarities to Kawasaki disease, with quick recognition and aggressive treatment with immunomodulatory therapies, MIS-C has been shown to be treatable with limited morbidity and mortality [3,4]. Throughout the last 2–3 years, numerous studies have described patient demographics, presenting symptoms, initial laboratory findings, cardiac findings, treatment algorithms, and short-term outcomes. However, what is still unclear about MIS-C is how patients do in the longer term with respect to lab abnormalities, inflammatory profiles, and cardiac sequelae. In this study, a large single center cohort of MIS-C patients is described, stratified by disease severity, and followed longitudinally to help define the natural history of MIS-C in the longer term. 

## 2. Materials and Methods

All patients less than or equal to 18 years of age hospitalized with MIS-C at Children’s of Alabama hospital in Birmingham, Alabama were identified between 1 March 2020 and 1 April 2022. Patients were identified from the electronic medical record using *International Classification of Diseases, 10th Revision* (ICD-10) codes for MIS-C (ICD-10 U07.1, M35.81) and then individually verified using the US Centers for Disease Control and Prevention (CDC) case definition for MIS-C. Clinical notes and the electronic medical record were reviewed for patient demographics, hospital course, imagining findings, and laboratory data.

Throughout the study period, MIS-C patients were classified as mild, moderate, or severe by the general pediatric and rheumatology services at Children’s of Alabama, for use in a standardized treatment algorithm. The treatment algorithm used at Children’s of Alabama is largely adapted from the American College of Rheumatology guidelines and leans towards early and aggressive treatment of inflammation with intravenous immunoglobulin (IVIG) and glucocorticoids as first line [5,6]. This same classification system was used to stratify the study population by severity.

1. Mild MIS-C patients are those who did not require significant fluid resuscitation, vasopressor medications, or positive pressure ventilation. Patients in this category received IVIG 2 g/kg once and were monitored for 36–48 h to determine if a low dose glucocorticoid course (prednisone or methylprednisolone 1–2 mg/kg/day tapered over 2–3 weeks) was needed for continued signs of inflammation.
2. Moderate MIS-C patients are those who required significant fluid resuscitation (up to 60 mL/kg) to maintain normotension and/or required a short course of low-dose vasopressor medications without requiring ventilation or suffering from organ failure. These patients received IVIG 2 g/kg and glucocorticoids at 1–2 mg/kg/day started on presentation, with glucocorticoids tapered over 2–3 weeks.
3. Severe MIS-C patients are those who required significant fluid resuscitation and high-dose/multiple vasopressor medications to maintain normotension, required positive pressure ventilation due to respiratory failure, had neurologic involvement with encephalopathy or seizures, and/or had organ failure on presentation. Severe patients were given IVIG 2 g/kg on admission and treated with high dose methylprednisolone (10–30 mg/kg/day, max 1000 mg daily) for 3–5 days with de-escalation thereafter to 1–2 mg/kg/day tapered over 2–3 weeks.

Patients’ laboratory data, imaging findings, and treatments were tracked throughout their hospitalization and at multi-disciplinary follow-up appointments at multiple defined time intervals: 1–2 weeks, 1–2 months, and 6–8 months post-hospitalization. Continuous variables are reported as medians and interquartile ranges (IQR), and categorical variables are summarized as frequencies and percentages. The proportions of abnormal lab values and cardiac findings at each time point were compared between severity groups, and statistical analysis was performed using the Chi-square test for independence at a *p*-value of 0.05. Median lab value comparisons were performed using the Kruskal–Wallis Test at a *p*-value of 0.05. Calculations were performed using Microsoft Excel.

## 3. Results

### 3.1. Demographics and Hospitalization Characteristics

137 MIS-C patients were identified during the study period and stratified into mild, moderate, and severe presentations, with 52, 44, and 41 patients in each group, respectively. Demographic information is noted in Table 1. Median age of all patients was 9 years of age, ranging from 8 weeks to 17 years, with age trending higher as severity increased. Male patients made up the majority of all groups and 64% of all patients. Black, non-Hispanic patients were in the majority of the total population at 55% and had even higher representation in the moderate and severe categories. Most MIS-C patients (74%) were healthy at baseline with no co-morbidities, with asthma being the most common pre-existing condition in the study population. Despite this lack of coexisting medical issues, the median body mass index (BMI) percentile of the study population was quite high, and severity tended to increase with BMI percentile.

Fever and gastrointestinal symptoms of nausea/vomiting, diarrhea, and abdominal pain were by far the most common presenting symptoms of the study population, followed by Kawasaki-like symptoms of rash, conjunctivitis, and mucosal changes. Neck pain, cervical lymphadenopathy, and headache were less common, but still seen in 20–30% of patients. Respiratory symptoms were less common still, only seen in 10–20% of patients. Encephalopathy was a rare presenting manifestation of MIS-C, but seen at a higher proportion in the severe classification.

40% of patients required intensive care unit admission for treatment of MIS-C, but the median length of stay for all patients was 5 days, only increased slightly at 6 days for severe patients. The vast majority (94–100%) of MIS-C patients tested positive for SARS-CoV-2 nucleocapsid IgG antibodies, with about a third also testing positive for SARS-CoV-2 via nasal polymerase chain reaction (PCR).

Vasopressor support was required in 37% of the study population and 93% of severe patients. The majority of patients did not require oxygen support or ventilation, with the need for both increasing with severity. All 137 patients in this study survived, with no mortality noted. 

### 3.2. Treatment

Treatments by severity classification are noted in Table 2. The vast majority of the study population received both glucocorticoids and IVIG as initial treatment of MIS-C, along with low dose aspirin as antiplatelet therapy per MIS-C guidelines. Higher severity categories were more likely to receive any glucocorticoids and higher dose glucocorticoids throughout their hospitalization. Only 4–5% of patients required second line treatment with infliximab or anakinra. About one-third of patients were given anticoagulation at some point during hospitalization—these patients usually were given enoxaparin in lieu of aspirin given severe septic shock or thrombocytopenia, then transitioned to aspirin once disease process and lab abnormalities had improved.

### 3.3. Laboratory Abnormalities and Improvement Trend

Full laboratory data by severity at admission, min/max, discharge, 1–2 week follow-up, 1–2 month follow-up, and 6–8 month follow-up time points can be found in Supplemental Materials Table S1. Laboratory testing that was tracked in the study population included sodium, creatinine, albumin, aspartate aminotransferase (AST), alanine transaminase (ALT), white blood cell (WBC) count, absolute lymphocyte count (ALC), hemoglobin (Hgb), platelet count, brain natriuretic peptide (BNP), troponin, C-reactive protein (CRP), erythrocyte sedimentation rate (ESR), ferritin, fibrinogen, and d-dimer.

Supplemental Figures S1 and S2  graph the proportions of patient with abnormal lab values over time by severity grouping. The proportion of patients with abnormal creatinine level, albumin level, WBC count, BNP, troponin, and fibrinogen were all found to be significantly different between severity groups at admission, during hospitalization, and/or at the time of discharge. *p*-values for significant proportional differences are seen in supplemental Figures S1 and S2 as well. However, the proportion of patients with abnormal sodium levels, AST, ALT, ALC, hemoglobin, platelet counts, d-dimer, CRP, ESR, and ferritin were similar between groups during the acute illness and at initial follow-up. At the 1–2 week follow-up appointment, all groups have similar proportions of abnormal labs. Furthermore, when all MIS-C patients are taken into account, there is statistically significant improvement in all lab findings between their minimum/maximum values and the 1–2 month follow-up period, as seen in Table 3.

### 3.4. Cardiac Outcomes

The main cardiac outcomes tracked in the study population were those seen on echocardiography at multiple time points throughout disease course. Cardiac data is seen in Table 4. 26% of all patients were noted to have depressed ejection fraction at initial cardiac echo, with the proportion of affected patients correlating with severity. The proportions of patients with decreased ejection fraction were significantly different at admission and discharge, but that difference disappeared by 1–2 month follow-up appointments. All patients screened with repeat echocardiography had complete normalization of ejection fraction and this improvement persisted to the 6–8 month follow-up.

Coronary artery abnormalities were defined as z-score > 2.0 and the reported z-score medians are reported for only those patients with documented dilation/aneurysm. Coronary artery changes on initial echocardiography were inversely related to severity of MIS-C, with the mild category having 4 (8%) patients affected compared with 1 (2%) severe patient, but this difference in proportion was not significant. However, by discharge from hospitalization, a higher raw number and percentage of severe patients were noted to have coronary changes with 6 (20%) patients affected, correlating with a statistically significant difference in proportions. Mild patients seemed to recover faster from coronary abnormalities, as only 1 patient was noted to have dilated coronaries by discharge. However, it is unclear if this is a true trend or a sampling error, as severe MIS-C patients were more likely to have follow-up echocardiograms during hospitalization. Regardless, by the 1–2 month follow-up appointment, all coronary dilation had resolved in the entire study population, a finding that persisted in those screened at the 6–8 month mark as well.

## 4. Discussion

The epidemiologic characteristics of our MIS-C population are relatively similar to previously described, large MIS-C cohorts, with respect to male predominance, median age, overrepresentation of racial and ethnic minorities, healthy baseline status, and similar presenting signs/symptoms [4,7,8].

At initial presentation of MIS-C, the patients in this study had a wide variety of laboratory abnormalities consistent with hyperinflammation and cardiac injury. MIS-C has been shown to cause hyponatremia, acute kidney injury, hypoalbuminemia, liver inflammation, lymphopenia with relative neutrophilia, anemia, early thrombocytopenia with late thrombocytosis, systemic inflammation with elevated CRP, ESR, and ferritin, cardiac injury and dysfunction with elevated BNP and troponin, and markers of coagulopathy [5]. These lab abnormalities are all seen to varying degrees in this reported cohort, some with clear correlation with disease severity and others abnormal regardless of category. 

Proportions of patients with abnormal creatinine, albumin, WBC count, BNP, troponin, and fibrinogen differed significantly between severity groupings at admission, during hospitalization and at discharge. Regardless, the vast majority of all patients had improvement and normalization of any lab abnormalities by the 1–2-week or 1–2 month follow-up appointment. However, it is unclear how well improvement in these values can be correlated to improvement in underlying inflammation. Sodium and albumin levels are complicated in their regulation with hydration, nutrition, and other factors playing a role. Similarly, WBC count and ALC can be influenced by presence of glucocorticoids, causing elevation in these levels. Although the proportion of patients with abnormalities in liver enzyme elevation, anemia, thrombocytopenia, and degree of CRP, ESR, and ferritin elevation were similar between severity groupings, the median values of these markers did seem to trend with disease severity (see Supplemental Table S1), with severe MIS-C patients having higher gross levels of these markers. Severe cases of MIS-C can present with distributive and hypotensive shock, along with higher levels of inflammatory chemokines/cytokines (CXCL9, IL-1, IL-6, IL-18, etc.), leading to higher likelihood of end-organ damage from hypo-perfusion and cytotoxicity [9,10]. Again, however, abnormalities in AST, ALT, and creatinine were all seen to resolve quickly regardless of disease severity. While CRP, anemia, and ferritin levels quickly improved in all groups regardless of initial degree of abnormality, ESR was slower to normalize. ESR can be elevated due to the use of IVIG therapy, so slower improvement in ESR is likely a combination of inflammatory improvement and long-term effect of IVIG. A study by Farooqi et al. showed similar improvement in 45 MIS-C patients with IVIG and steroid treatment by the 1–4 month follow-up period, with the majority of labs normalized in 80% of patients [11]. Correlating with basic laboratory abnormalities, Farooqi et al. also showed significant abnormalities in lymphocyte subsets at presentation which improved by the 1–4 month marks as well, showing correlation with improvement of underlying immune dysregulation with treatment [11]. Another study of 46 MIS-C patients by Penner et al. showed similar improvement in lab markers by 6 months [12]. These studies and the results herein show that the majority of lab abnormalities normalize by 6 months regardless of severity, some as early as 1–2 weeks or 1–2 months.

One of the most concerning effects of MIS-C is its potential for serious cardiac abnormalities. MIS-C was first identified due its similarity to Kawasaki disease with rash, conjunctivitis, and mucosal changes, but also with its effect on the coronary arteries and systolic function [13]. Decreased ejection fraction has been seen in 30–40% of MIS-C patients and coronary artery dilatation in 13–28% [4,7,14]. The cohort reported here had a somewhat lower incidence of both decreased ejection fraction and coronary changes, but the severe classifications were similar in their cardiac findings. The proportion of patients with abnormalities in BNP and troponin differed significantly between severity groups during hospitalization. These severe MIS-C patients also seem to have more drastic elevation in BNP and troponin correlating with their echocardiography findings, with troponin remaining abnormal in the majority of severe MIS-C patient during hospitalization. The majority of all patients developed elevation in BNP at some point during hospitalization, but severe patients seemed to have higher levels of BNP at initial presentation. Fluid resuscitation has a profound effect on heart stretch and can raise BNP levels, even with normal underlying cardiac function. Therefore, BNP on presentation is likely most helpful in determining baseline cardiac dysfunction. However, both BNP and troponin rapidly normalized in the outpatient setting across the board. 

In this study, there was found to be a significant difference in proportions of patients with abnormal cardiac ejection fraction at admission and discharge, and in coronary artery changes at discharge. However, by 1–2 month follow-up appointments, all patients had resolution of these cardiac abnormalities. Previous studies have demonstrated similar rapid improvement in cardiac abnormalities after appropriate treatment with IVIG and glucocorticoids. In one study of 50 MIS-C patients, 52% had coronary abnormalities in the acute period, decreasing to 27% at 2 weeks, and all resolving by 6 months [15]. 52% of this same cohort had decreased left ventricular ejection fraction at presentation, all resolved by 8 weeks [15]. Farooqi et al. similarly showed 48.9% with decreased ejection fraction at presentation, 10.3% abnormal at 1–4 weeks, and all resolved by 4–9 months [11]. Even when evaluated with more sensitive cardiac magnetic resonance imaging (MRI), cardiac abnormalities show similar improvement. A study of 19 consecutive MIS-C patients, 18 of which had decreased ejection fraction during the acute disease process, showed complete resolution in all patients at a median follow-up of 99 days (IQR 89-104) [16]. It is clear that with appropriate treatment, even severe cardiac abnormalities caused by MIS-C will rapidly resolve. 

The present study does have some limitations. First, this study is largely retrospective in nature and although there are quite a few MIS-C patients, it is smaller than some of the more impressive population studies. Second, although the treatment algorithm was largely adhered to during the COVID-19 pandemic, there were some changes that took place during the study period. Early into the experience with MIS-C, it was more common to allow higher volumes of fluid resuscitation prior to vasopressor initiation and there was more hesitation to start early steroid use given the unfamiliarity with the disease process. As the collective experience grew, early vasopressor and glucocorticoid use became the norm, to prevent issues related to fluid overload and pulmonary edema, and to prevent need for higher pulse doses or second line therapy, respectively. This may have confounded the data somewhat, with improved outcomes in later patients. Finally, although standardized follow-up was attempted, there was decent variation in follow-up timing and missed visits, which could lead to skewed data in some cases. For instance, out of 137 total patients, only 40–50 patients were seen at the 6–8 month follow-up appointment, making generalizability questionable and making it difficult to draw definitive conclusions. 

## 5. Conclusions

Although MIS-C is a serious, post-infectious consequence of SARS-CoV-2 infection in children, patients overall do very well with rapid diagnosis and aggressive treatment. When treated appropriately, laboratory abnormalities, coronary dilatation, and systolic dysfunction will improve rapidly within 6 months or sooner in most cases. Providers should continue to be mindful of MIS-C as we shift into new phases of the COVID-19 pandemic, in order to prevent the most dangerous complications of this novel disease. 

## Figures and Tables

**Table 1:** Demographics.

	All n = 137	Mild n = 52	Moderate n = 44	Severe n = 41
Demographics				
Age, years—median (IQR)	9 (5–12)	8 (4–11.3)	9.5 (6–13)	11 (7–12)
Male sex—no. (%)	88 (64%)	33 (63%)	28 (64%)	27 (66%)
Race/Ethnicity—no. (%)				
White, non-Hispanic	48 (35%)	25 (48%)	13 (30%)	10 (24%)
Black, non-Hispanic	75 (55%)	21 (40%)	27 (61%)	27 (66%)
Hispanic	12 (9%)	6 (12%)	3 (7%)	3 (7%)
Other	2 (1%)	0 (0%)	1 (2%)	1 (2%)
BMI percentile–median (IQR)	84 (41–97)	79.5 (27.3–96.3)	87.5 (56–97.3)	89 (66–98)
Co-morbidities—no. (%)				
None	101 (74%)	36 (69%)	31 (70%)	34 (83%)
Any	36 (26%)	16 (31%)	13 (30%)	7 (17%)
Asthma	25	12	9	4
Autism	3	-	3	-
Congenital Heart Disease	2	1	1	-
Hypertension	2	1	-	1
Leukemia/Lymphoma in remission	1	1	-	-
Precocious Puberty	1	1	-	-
Ex-29 wga w/chronic lung dx	1	-	-	1
Type 1 Diabetes	1	-	-	1
Symptoms on Presentation—no. (%)				
Fever	134 (98%)	52 (100%)	44 (100%)	38 (93%)
Respiratory Symptoms	27 (20%)	15 (29%)	6 (14%)	6 (15%)
Cough	23 (17%)	14 (27%)	5 (11%)	4 (10%)
Shortness of Breath	10 (7%)	3 (6%)	3 (7%)	4 (10%)
Gastrointestinal Symptoms	124 (91%)	43 (83%)	41 (93%)	40 (98%)
Nausea/Vomiting	92 (67%)	32 (62%)	28 (64%)	32 (78%)
Diarrhea	62 (45%)	17 (33%)	22 (50%)	23 (56%)
Abdominal Pain	84 (61%)	25 (48%)	33 (75%)	26 (63%)
Rash	73 (53%)	32 (62%)	23 (52%)	18 (44%)
Conjunctivitis	86 (63%)	35 (67%)	26 (59%)	25 (61%)
Mucosal Changes	54 (39%)	19 (37%)	17 (39%)	18 (44%)
Neck pain/Cervical Lymphadenopathy	46 (34%)	15 (29%)	17 (39%)	14 (34%)
Headache	40 (29%)	16 (31%)	12 (27%)	12 (29%)
AMS or Encephalopathy	10 (7%)	3 (6%)	1 (2%)	6 (15%)
Level of Admission—no. (%)				
Intensive Care	55 (40%)	0 (0%)	20 (45%)	35 (85%)
ICU step-down unit	26 (19%)	13 (25%)	10 (23%)	3 (7%)
Acute Care	56 (41%)	39 (75%)	14 (32%)	3 (7%)
Length of stay, days—median (IQR)	5 (4–6)	4 (4–6)	5 (4.8–6)	6 (5–8)
SARS-CoV-2 Positivity				
PCR—no./total no. (%)	42/135 (31%)	14/50 (28%)	16 (36%)	12 (29%)
IgG Antibodies—no./total no. (%)	130/135 (96%)	49 (94%)	42 (95%)	39/39 (100%)
Vasopressor Requirement—no. (%)				
Yes	51 (37%)	0 (0%)	13 (30%)	38 (93%)
No	86 (63%)	52 (100%)	31 (70%)	3 (7%)
Respiratory Support—no. (%)				
None	77 (56%)	47 (90%)	21 (48%)	9 (22%)
Oxygen—Nasal Cannula	40 (29%)	4 (8%)	20 (45%)	16 (39%)
Oxygen—High-Flow Nasal Cannula	10 (7%)	1 (2%)	3 (7%)	6 (15%)
Positive pressure ventilation	10 (7%)	0 (0%)	0 (0%)	10 (24%)
Survival—no. (%)	137 (100%)	52 (100%)	44 (100%)	41 (100%)

Abbreviations: no.—number; BMI—body mass index; wga—weeks gestational age; AMS—altered mental status; ICU—intensive care unit; PCR—polymerase chain reaction; IgG—immunoglobulin G.

**Table 2:** Treatment.

	All n = 137	Mild n = 52	Moderate n = 44	Severe n = 41
Initial Therapy				
Glucocorticoids—no. (%)	117 (85%)	34 (65%)	43 (98%)	40 (98%)
Initial dose, mg/kg/day-median (IQR)	2 (2–12.5)	2 (1.7–2)	2 (2–13.75)	2 (2–20)
Maximum dose, mg/kg/day—median (IQR)	2 (2–15)	2 (2–2)	2 (2–15.5)	10 (2–20)
Intravenous Immunoglobulin—no. (%)	133 (97%)	50 (96%)	44 (100%)	39 (95%)
Second-line Therapy				
Anakinra—no. (%)	7 (5%)	1 (2%)	0 (0%)	6 (15%)
Infliximab-—no. (%)	6 (4%)	4 (8%)	2 (5%)	0 (0%)
Anticoagulation/Antiplatelet Therapy				
Enoxaparin—no. (%)	45 (33%)	1 (2%)	18 (41%)	26 (63%)
Aspirin—no. (%)	134 (98%)	52 (100%)	44 (100%)	38 (93%)

**Table 3:** Improvement in laboratory findings in all patients.

	Max/Min Value		1-2-Week f/u		*p*-Value
	n	Median	n	Median	
Sodium (mmol/L)	136	132	98	137	<0.00001
Creatinine (mg/dL)	136	0.685	98	0.465	<0.00001
Albumin (g/dL)	136	2.5	98	3.9	<0.00001
AST (U/L)	133	47	97	27	<0.00001
ALT (U/L)	133	41.3	97	30.8	0.00857
White Blood Cell count (10^3^/uL)	134	18.04	98	7.35	<0.00001
Absolute Lymphocyte count (10^3^/uL)	133	0.86	98	2.47	<0.00001
Hemoglobin (g/dL)	134	8.95	98	11.7	<0.00001
Platelet count (10^3^/uL)	134	174 (Min)	98	400	<0.00001
Brain Natriuetic Peptide (pg/mL)	118	798.1	81	10	<0.00001
Troponin (ng/mL)	97	0.13	60	0.01	<0.00001
Fibrinogen (mg/dL)	95	591 (Max)	61	292	<0.00001
D-Dimer DDU (ng/mL)	93	1915	75	245	<0.00001
CRP (mg/dL)	135	20.25	97	0.1	<0.00001
ESR (mm/hr)	121	51 (Initial)	59	30	0.00003
Ferritin (ng/mL)	133	693	93	143.7	<0.00001

**Table 4:** Cardiac Outcomes.

	Admission			Discharge			1–2 Month Follow-Up		
	n	Percent Abnormal	Median (IQR)	n	Percent Abnormal	Median (IQR)	n	Percent Abnormal	Median (IQR)
Decreased Ejection Fraction-(normal > 55%)									
ALL	137	26% n = 36	60% (53.3–68%) n = 137	80	13% n = 10	63.5% (58.8–68%) n = 80	103	0% n = 0	67% (63.5–71%) n = 103
Mild	52	6% n = 3	65% (60–69%) n = 52	20	0% n = 0	66% (63.5–69%) n = 20	41	0% n = 0	68% (63–72%) n = 41
Moderate	44	25% n = 11	59% (54.5–65%) n = 44	30	10% n = 3	60.5% (57.8–67.3%) n = 30	35	0% n = 0	67% (65–70.3%) n = 35
Severe	41	54% n = 22	52% (45–66%) n = 41	30	23% n = 7	63% (56–67%) n = 30	27	0% n = 0	66.5% (62.5–69%) n = 27
*p*-value—3-group comparison		1.24 × 10^-6^			0.044			N/A	
Coronary Artery dilation/aneurysm-max z-score									
ALL	137	6% n = 8	2.38 (2.16–2.91) n = 8	80	10% n = 8	2.18 (2.10–2.50) n = 8	103	0% n = 0	-
Mild	52	8% n = 4	2.81 (2.34–3.28) n = 4	20	2% n = 1	2.36 n = 1	41	0% n = 0	-
Moderate	44	7% n = 3	2.35 (2.23–2.58) n = 3	30	3% n = 1	2.12 n = 1	35	0% n = 0	-
Severe	41	2% n = 1	2.15 n = 1	30	20% n = 6	2.17 (2.09–2.74) n = 6	27	0% n = 0	-
*p*-value—3-group comparison		0.532			0.068			N/A	

## Data Availability

Data available upon request to corresponding author.

## Supplementary Materials

The following supporting information can be downloaded at: https://www.mdpi.com/article/10.3390/v15010094/s1, Table S1: Full Laboratory Data, Figure S1, Figure S2.

## References

[1] Reiff D.D., Cron R.Q. (2022). Who Would Have Predicted Multisystem Inflammatory Syndrome in Children?. Curr. Rheumatol. Rep..

[2] Centers for Disease Control and Prevention: Multisystem Inflammatory Syndrome. https://www.cdc.gov/mis/index.html.

[3] Cattalini M., Della Paolera S., Zunica F., Bracaglia C., Giangreco M., Verdoni L., Meini A., Sottile R., Caorsi R., Zuccotti G. (2021). Defining Kawasaki disease and pediatric inflammatory multisystem syndrome-temporally associated to SARS-CoV-2 infection during SARS-CoV-2 epidemic in Italy: Results from a national, multicenter survey. Pediatr. Rheumatol..

[4] Feldstein L.R., Tenforde M.W., Friedman K.G., Newhams M., Rose E.B., Dapul H., Soma V.L., Maddux A.B., Mourani P.M., Bowens C. (2021). Characteristics and Outcomes of US Children and Adolescents with Multisystem Inflammatory Syndrome in Children (MIS-C) Compared with Severe Acute COVID-19. JAMA.

[5] Henderson L.A., Canna S.W., Friedman K.G., Gorelik M., Lapidus S.K., Bassiri H., Behrens E.M., Kernan K.F., Schulert G.S., Seo P. (2022). American College of Rheumatology Clinical Guidance for Multisystem Inflammatory Syndrome in Children Associated with SARS-CoV-2 and Hyperinflammation in Pediatric COVID-19: Version 3. Arthritis Rheumatol..

[6] Ouldali N., Toubiana J., Antona D., Javouhey E., Madhi F., Lorrot M., Léger P.-L., Galeotti C., Claude C., Wiedemann A. (2021). Association of Intravenous Immunoglobulins Plus Methylprednisolone vs Immunoglobulins Alone with Course of Fever in Multisystem Inflammatory Syndrome in Children. JAMA.

[7] Feldstein L.R., Rose E.B., Horwitz S.M., Collins J.P., Newhams M.M., Son M.B.F., Newburger J.W., Kleinman L.C., Heidemann S.M., Martin A.A. (2020). Multisystem Inflammatory Syndrome in U.S. Children and Adolescents. N. Engl. J. Med..

[8] Valverde I., Singh Y., Sanchez-De-Toledo J., Theocharis P., Chikermane A., Di Filippo S., Kuciñska B., Mannarino S., Tamariz-Martel A., Gutierrez-Larraya F. (2021). Acute Cardiovascular Manifestations in 286 Children with Multisystem Inflammatory Syndrome Associated With COVID-19 Infection in Europe. Circulation.

[9] Chang J.C., Matsubara D., Morgan R.W., Diorio C., Nadaraj S., Teachey D.T., Bassiri H., Behrens E.M., Banerjee A. (2021). Skewed Cytokine Responses Rather Than the Magnitude of the Cytokine Storm May Drive Cardiac Dysfunction in Multisystem Inflammatory Syndrome in Children. J. Am. Heart Assoc..

[10] Rodriguez-Smith J.J., Verweyen E.L., Clay G.M., Esteban Y.M., de Loizaga S.R., Baker E.J., Do T., Dhakal S., Lang S.M., A Grom A. (2021). Inflammatory biomarkers in COVID-19-associated multisystem inflammatory syndrome in children, Kawasaki disease, and macrophage activation syndrome: A cohort study. Lancet Rheumatol..

[11] Farooqi K.M., Chan A., Weller R.J., Mi J., Jiang P., Abrahams E., Ferris A., Krishnan U.S., Pasumarti N., Suh S. (2021). Longitudinal Outcomes for Multisystem Inflammatory Syndrome in Children. Pediatrics.

[12] Penner J., Abdel-Mannan O., Grant K., Maillard S., Kucera F., Hassell J., Eyre M., Berger Z., Hacohen Y., Moshal K. (2021). 6-month multidisciplinary follow-up and outcomes of patients with paediatric inflammatory multisystem syndrome (PIMS-TS) at a UK tertiary paediatric hospital: A retrospective cohort study. Lancet Child Adolesc. Health.

[13] Riphagen S., Gomez X., Gonzalez-Martinez C., Wilkinson N., Theocharis P. (2020). Hyperinflammatory shock in children during COVID-19 pandemic. Lancet.

[14] Haghighi Aski B., Manafi Anari A., Abolhasan Choobdar F., Zareh Mahmoudabadi R., Sakhaei M. (2021). Cardiac abnormalities due to multisystem inflammatory syndrome temporally associated with Covid-19 among children: A systematic review and meta-analysis. Int. J. Cardiol. Heart Vasc..

[15] Capone C.A., Subramony A., Sweberg T., Schneider J., Shah S., Rubin L., Schleien C., Epstein S., Johnson J.C., Kessel A. (2020). Characteristics, cardiac involvement, and outcomes of multisystem infammatory syndrome of childhood associated with severe acute respiratory syndrome coronavirus 2 Infection. J. Pediatr..

[16] Bartoszek M., Małek Ł.A., Barczuk-Falęcka M., Brzewski M. (2022). Cardiac Magnetic Resonance Follow-Up of Children After Pediatric Inflammatory Multisystem Syndrome Temporally Associated with SARS-CoV-2 with Initial Cardiac Involvement. J. Magn. Reason. Imaging.

