# Supplementary material for: Timely Resolution of SARS-CoV-2-Related Multi-System Inflammatory Syndrome in Children
Source: Viruses. 2022 Dec 29;15(1):94. doi: 10.3390/v15010094 (PMC9866445; doi:10.3390/v15010094)
Supplement: Supplementary file 1 [file viruses-15-00094-s001.zip › Supplmentary Table 1.pdf]

Supplemental Table 1. Laboratory Values

	Admission			Minimum/Maximum			Discharge			1-2 week Follow-up			1-2 month Follow-up			6-8 month follow-up		
	n	Percent abnormal	Median	n	Percent abnormal	Median	n	Percent abnormal	Median	n	Percent abnormal	Median	n	Percent abnormal	Median	n	Percent abnormal	Median
Sodium (138-145 mmol/L)						***MIN***												
ALL	137	86.9%	134 (132-136)	136	98.5%	132 (130-134)	133	66.9%	137 (135-138)	98	52.0%	137 (136.3-139)	99	20.2%	139 (138-140)	54	11.1%	139 (138-141)
Mild	52	84.6%	134 (132-136)	51	96.1%	132 (130-135)	48	75.0%	136 (135-137.3)	34	58.8%	137 (136-139)	39	15.4%	139 (138-140)	20	10.0%	139.5 (138-141)
Moderate	44	90.9%	134 (132-136)	44	100.0%	133 (131-134)	44	70.5%	137 (135-138)	36	55.6%	137 (136-139)	33	33.3%	139 (137-140)	21	19.0%	139 (138-140)
Severe	41	85.4%	133 (130-135)	41	100.0%	131 (129-133)	41	53.7%	137 (136-138)	28	39.3%	139 (137-140)	27	11.1%	139 (138-140)	13	0.0%	140 (139-141)
Creatinine (0.2-0.8 mg/dL)						***MAX***												
ALL	137	34.3%	0.66 (0.47-0.9)	136	36.8%	0.69 (0.52-0.93)	133	4.5%	0.42 (0.33-0.52)	98	4.1%	0.47 (0.35-0.59)	99	5.1%	0.48 (0.39-0.59)	54	13.0%	0.53 (0.41-0.66)
Mild	52	7.7%	0.53 (0.4-0.67)	51	7.8%	0.54 (0.41-0.69)	48	0.0%	0.38 (0.32-0.5)	34	0.0%	0.4 (0.3-0.54)	39	2.6%	0.49 (0.38-0.54)	20	0.0%	0.46 (0.39-0.54)
Moderate	44	38.6%	0.68 (0.5-0.9)	44	40.9%	0.69 (0.52-0.9)	44	4.5%	0.415 (0.34-0.54)	36	2.8%	0.47 (0.36-0.64)	33	6.1%	0.5 (0.41-0.64)	21	9.5%	0.57 (0.41-0.69)
Severe	41	63.4%	0.9 (0.69-1.9)	41	68.3%	1.05 (0.71-2.3)	41	9.8%	0.45 (0.39-0.55)	28	10.7%	0.49 (0.4-0.56)	27	7.4%	0.46 (0.39-0.56)	13	38.5%	0.6 (.41-0.87)
Albumin (3.8-4.8 g/dL)						***MIN***												
ALL	137	76.6%	3.4 (2.9-3.7)	136	100.0%	2.5 (2.2-2.7)	133	97.0%	2.7 (2.6-3.1)	98	18.4%	3.9 (3.8-4.1)	99	3.0%	4.1 (4-4.3)	54	3.7%	4.3 (4.1-4.4)
Mild	52	63.5%	3.6 (3.2-3.9)	51	100.0%	2.6 (2.5-2.9)	48	95.8%	2.95 (2.7-3.1)	34	14.7%	3.9 (3.8-4.1)	39	2.6%	4.2 (4-4.3)	20	10.0%	4.2 (4.1-4.4)
Moderate	44	84.1%	3.4 (3.1-3.6)	44	100.0%	2.5 (2.3-2.6)	44	100.0%	2.7 (2.6-2.9)	36	13.9%	4 (3.9-4.1)	33	6.1%	4.1 (4-4.2)	21	0.0%	4.3 (4.1-4.4)
Severe	41	85.4%	2.9 (2.5-3.4)	41	100.0%	2.3 (2-2.5)	41	95.1%	2.6 (2.4-3.1)	28	28.6%	3.9 (3.7-4.2)	27	0.0%	4.1 (4-4.3)	13	0.0%	4.3 (4.1-4.4)
AST (15-40 U/L)						***MAX***												
ALL	136	51.5%	41.5 (25-56.3)	133	60.9%	47 (31-70)	126	31.0%	31 (24.3-43.8)	97	11.3%	27 (21-35)	99	4.0%	24 (19-29.5)	54	0.0%	23 (18-26)
Mild	51	47.1%	31 (23-53.5)	50	50.0%	40 (29-58.3)	47	21.3%	27 (24-38.5)	34	5.9%	27 (21.5-35.8)	39	2.6%	24 (18-30)	20	0.0%	24.5 (19-27.5)
Moderate	44	52.3%	41 (30.8-55.3)	44	63.6%	46.5 (37.5-65.5)	43	37.2%	34 (24.5-45)	36	13.9%	29.5 21-35.3)	33	0.0%	25 (19-30)	21	0.0%	23 (21-25)
Severe	41	56.1%	43 (31-72)	39	71.8%	54 (36.5-79.5)	36	36.1%	34.5 (26.8-50)	27	14.8%	26 (23-33)	27	11.1%	24 (21.5-27.5)	13	0.0%	17 (16-25)
ALT (9-25 U/L)						***MAX***												
ALL	136	62.5%	32.8 (18.6-55.9)	133	75.2%	41.3 (25.4-66.3)	126	61.1%	32.8 (20.7-50)	97	62.9%	30.8 (21.1-46.4)	99	17.2%	16.5 (12.9-20.4)	54	13.0%	16.3 (12.2-19.3)

Mild	51	52.9%	26.9 (13.7-52.9)	50	66.0%	34.4 (18.8-62.2)	47	51.1%	25.4 (15.6-36.3)	34	58.8%	29 (20.3-39.1)	39	17.9%	15.5 (11.8-19.7)	20	20.0%	17.2 (11.5-21.8)
Moderate	44	63.6%	35.6 (20.5-53.8)	44	75.0%	47.7 (25.5-65.7)	43	65.1%	38.7 (23-50)	36	69.4%	39.3 (23.3-52.4)	33	15.2%	18.2 (13.9-20.7)	21	4.8%	14.5 (12.6-18.2)
Severe	41	73.2%	34.7 (24.9-62.6)	39	87.2%	44 (31.8-70.4)	36	69.4%	36.5 (22.5-61.9)	27	59.3%	28 (18.2-59.7)	27	18.5%	16 (12.9-21.3)	13	15.4%	16.8 (13.5-20.2)
White Blood Cell count (4.0-11.0 x10^3/uL)						***MAX***												
ALL	137	52.6%	10.86 (7.84-14.53)	134	84.3%	18.04 (12.22-23.85)	134	73.9%	14.54 (10.21-19.82)	98	24.5%	7.35 (6.0-10.93)	99	11.1%	5.86 (4.7-7.42)	55	18.2%	6.09 (4.63-7.71)
Mild	52	50.0%	10.63 (7.45-14.12)	50	76.0%	14.46 (10.8-20.14)	50	64.0%	12.31 (8.87-16.66)	33	27.3%	7.76 (6.15-11.16)	39	0.0%	6.69 (5.59-7.82)	21	9.5%	6.19 (4.69-8.31)
Moderate	44	38.6%	9.58 (7.39-11.95)	44	84.1%	17.69 (11.9-21.78)	43	72.1%	14.69 (10.56-17.77)	37	21.6%	7.48 (6.01-10.79)	33	18.2%	5.16 (4.45-7.13)	21	33.3%	5.56 (3.94-6.61)
Severe	41	70.7%	12.73 (10.08-16.56)	40	95.0%	22.54 (18.31-32.08)	41	87.8%	18.31 (13.23-27.54)	28	25.0%	6.79 (5.95-10.04)	27	18.5%	5.45 (4.22-6.43)	13	7.7%	6.39 (4.8-7.9)
Absolute Lymphocyte count (1.13-5.52 x10^3/uL)						***MIN***												
ALL	136	50.7%	1.1 (0.76-1.7)	133	66.9%	0.86 (0.63-1.31)	133	8.3%	2.51 (1.63-3.91)	98	6.1%	2.47 (1.92-3.45)	99	1.0%	2.41 (1.9-2.96)	55	0.0%	2.21 (1.95-2.85)
Mild	52	42.3%	1.22 (0.88-2.04)	50	60.0%	0.98 (0.68-1.66)	50	12.0%	2.24 (1.53-3.69)	33	3.0%	3.05 (2.29-3.48)	39	2.6%	2.59 (2.18-3.18)	21	0.0%	2.25 (2.15-3.09)
Moderate	44	54.5%	1.05 (0.69-1.35)	43	74.4%	0.79 (0.59-1.13)	42	7.1%	2.35 (1.66-3.43)	37	5.4%	2.5 (1.92-3.68)	33	0.0%	2.24 (1.81-2.84)	21	0.0%	2.24 (1.91-2.81)
Severe	40	57.5%	1.03 (0.69-1.62)	40	67.5%	0.86 (0.59-1.28)	41	4.9%	2.78 (1.72-4.22)	28	10.7%	2.26 (1.68-3.2)	27	0.0%	2.24 (1.85-2.78)	13	0.0%	1.96 (1.8-2.29)
Hemoglobin (10-13 g/dL)						***MIN***												
ALL	137	20.4%	11.1 (10.1-12.2)	134	76.9%	9.0 (8.1-9.9)	134	41.0%	10.3 (9.2-11.1)	98	7.1%	11.7 (10.9-12.3)	99	1.0%	12.4 (11.8-12.8)	55	0.0%	13 (12.3-13.8)
Mild	52	17.3%	11.5 (10.5-12.3)	50	62.0%	9.6 (8.4-10.4)	50	42.0%	10.3 (9.6-11)	33	6.1%	11.5 (10.9-12.2)	39	2.6%	12.2 (11.9-12.7)	21	0.0%	13.1 (12.6-13.7)
Moderate	44	15.9%	11.2 (10.5-12.2)	44	81.8%	9.2 (8.5-9.8)	43	32.6%	10.2 (9.7-11.3)	37	8.1%	11.9 (11.4-12.5)	33	0.0%	12.5 (12.3-12.8)	21	0.0%	13 (12.2-14.1)
Severe	41	29.3%	10.4 (9.7-11.3)	40	90.0%	8.4 (7.6-9.1)	41	48.8%	10 (8.6-10.8)	28	7.1%	11.3 (10.6-11.9)	27	0.0%	12 (11-12.8)	13	0.0%	12.8 (12-13.1)
Platelet count (140-440 x10^3/uL)					***MIN/MAX***	***MIN/MAX***												
ALL	137	33.6%	186 (131-248)	134	39.6% / 41.8%	174 / 372 (118-225) / (282-485)	134	41.8%	356 (178-479)	98	39.8%	400 (307-496)	99	16.2%	349 (295-409)	55	7.3%	306 (266-377)
Mild	52	28.8%	228 (152-282)	50	40% / 50%	207 / 372.5 (120-254) / (257-484)	50	50.0%	371 (257-479)	33	39.4%	396 (348-533)	39	15.4%	365 (304-427)	21	14.3%	303 (270-384)
Moderate	44	29.5%	179 (138-228)	44	36.4% / 36.4%	170 / 376 (126-198) / (292-456)	43	37.2%	344 (294-457)	37	43.2%	398 (295-517)	33	9.1%	338 (282-374)	21	0.0%	306 (269-339)
Severe	41	43.9%	159 (10-213)	40	42.5% / 37.5%	154 / 345 (106-205) / (261-551)	41	36.6%	349 (229-549)	28	35.7%	401.5 (277-466)	27	25.9%	354 (296-425)	13	7.7%	307 (259-389)
Brain Natriuetic Peptide (7-30 pg/mL)						***MAX***												

ALL	134	74.6%	235.7 (28.8-962.6)	118	95.8%	798.1 (270.8-1679.3)	99	92.9%	248.6 (106.4-528.5)	81	11.1%	10 (10-17.3)	97	3.1%	10 (10-10)	54	7.4%	10 (10-14.6)
Mild	50	54.0%	35.9 (10-135.8)	39	89.7%	245.1 (121.5-780.9)	30	83.3%	175.8 (56.2-385.3)	23	8.7%	10 (10-15.3)	38	7.9%	10 (10-10.5)	21	14.3%	10.6 (10-15.2)
Moderate	44	84.1%	311.4 (75.6-878.7)	41	100.0%	644.7 (350.5-1207)	37	97.3%	232.3 (141.3-422.3)	35	11.4%	10 (10-19.6)	33	0.0%	10 (10-10)	21	4.8%	10 (10-11.3)
Severe	40	90.0%	1018.6 (274.7-2591.7)	38	97.4%	1835.1 (999.2-3150)	32	96.9%	378.8 (150.7-782.2)	23	13.0%	13.4 (10-21.7)	26	0.0%	10 (10-10)	12	0.0%	10 (10-14.6)
Troponin (<0.05 ng/mL)						***MAX***												
ALL	134	44.8%	0.03 (0.01-0.26)	97	67.0%	0.13 (0.03-0.49)	72	48.6%	0.05 (0.01-0.13)	60	6.7%	0.01 (0.01-0.01)	96	2.1%	0.01 (0.01-0.01)	52	0.0%	0.01 (0.01-0.01)
Mild	49	16.3%	0.01 (0.01-0.02)	31	38.7%	0.03 (0.01-0.25)	21	33.3%	0.01 (0.01-0.06)	14	0.0%	0.01 (0.01-0.01)	37	2.7%	0.01 (0.01-0.01)	20	0.0%	0.01 (0.01-0.01)
Moderate	44	47.7%	0.05 (0.01-0.18)	31	71.0%	0.11 (0.05-0.26)	27	37.0%	0.04 (0.02-0.09)	29	6.9%	0.01 (0.01-0.01)	32	3.1%	0.01 (0.01-0.01)	20	0.0%	0.01 (0.01-0.01)
Severe	41	75.6%	0.28 (0.07-0.61)	35	88.6%	0.38 (0.14-1.02)	24	75.0%	0.18 (0.06-0.47)	17	11.8%	0.01 (0.01-0.02)	27	0.0%	0.01 (0.01-0.01)	12	0.0%	0.01 (0.01-0.01)
CRP (0.0-0.5 mg/dL)						***MAX***												
ALL	137	100.0%	17.2 (12.4-23.5)	135	100.0%	20.3 (14.5-24.8)	134	98.5%	3.9 (2.5-7.6)	97	11.3%	0.1 (0.1-0.3)	97	12.4%	0.1 (0-0.2)	54	13.0%	0.1 (0-0.2)
Mild	52	100.0%	15.6 (11.3-19.5)	51	100.0%	18.2 (11.6-22.4)	50	98.0%	4.3 (3.1-9.8)	33	15.2%	0.1 (0.1-0.3)	38	15.8%	0.1 (0-0.2)	21	14.3%	0.1 (0-0.2)
Moderate	44	100.0%	17.14 (12.3-23.1)	44	100.0%	20.2 (14.7-25.0)	44	97.7%	4.4 (2.5-7.6)	37	13.5%	0.1 (0.1-0.2)	32	15.6%	0 (0-0.1)	21	4.8%	0.1 (0-0.1)
Severe	41	100.0%	22.4 (14.9-26.4)	40	100.0%	23.8 (18.6-28.7)	40	100.0%	3.0 (1.6-5.3)	27	3.7%	0.1 (0.1-0.2)	27	3.7%	0 (0-0.1)	12	25.0%	0.2 (0-0.5)
ESR (0-15 mm/hr)																		
ALL	121	98.3%	51 (37-70)	-	-	-	-	-	-	59	83.1%	30 (19-56)	97	44.3%	13 (9-23)	53	11.3%	8 (7-12)
Mild	45	97.8%	47 (32-62)	-	-	-	-	-	-	17	88.2%	28 (20-65)	37	54.1%	17 (11-25)	20	5.0%	8 (7-10)
Moderate	39	97.4%	48 (40-70)	-	-	-	-	-	-	25	80.0%	34 (19-47)	33	33.3%	12 (8-18)	21	4.8%	8 (6-9)
Severe	37	100.0%	58 (45-76)	-	-	-	-	-	-	17	82.4%	29 (21-46)	27	44.4%	12 (10-28)	12	33.3%	13 (7-16)
Ferritin (5.3-99.9 ng/mL)						***MAX***												
ALL	134	97.0%	527.4 (297.8-868.1)	133	100.0%	693 (416.4-1344.6)	127	100.0%	438.7 (270.5-719.4)	93	68.8%	143.7 (82-223)	97	10.3%	40.2 (25.5-63)	54	1.9%	25.1 (20.1-37.8)
Mild	51	94.1%	365.6 (205.6-635.4)	49	100.0%	603.7 (318.4-851.9)	46	100.0%	450.8 (239.5-728.4)	31	67.7%	138.5 (68.4-211.2)	39	7.7%	37.9 (24.9-64.6)	21	0.0%	25.9 (22.7-41.3)
Moderate	43	97.7%	472.4 (322.5-852.1)	44	100.0%	615.3 (459.6-1064)	43	100.0%	399.7 (268.5-556.4)	36	63.9%	144.7 (81.7-234.0)	33	9.1%	37.8 (28.3-64.7)	21	0.0%	24.3 (21.5-36.4)

Severe	40	100.0%	814.8 (549.3-1823.2)	40	100.0%	1139 (665.1-2452.3)	38	100.0%	508.6 (376.8-789.2)	26	76.9%	141.7 (106.4-238.3)	25	16.0%	40.7 (28.3-61.5)	12	8.3%	26.8 (16.1-37.8)
Fibrinogen (156-400 mg/dL)						***MAX***												
ALL	128	87.5%	554 (462-673)	95	87.4%	591 (450-705)	77	48.1%	390 (298-517)	61	8.2%	292 (253-322)	67	11.9%	321 (285-361)	52	11.5%	289 (256-350)
Mild	49	91.8%	544 (450-652)	36	91.7%	594 (440-687)	29	65.5%	414 (368-538)	15	13.3%	287 (255-320)	27	18.5%	348 (283-384)	20	5.0%	274 (249-337)
Moderate	43	83.7%	566 (460-666)	33	81.8%	566 (477-673)	27	48.1%	361 (282-505)	29	6.9%	308 (271-327)	23	13.0%	311 (283-349)	20	10.0%	303 (268-336)
Severe	36	86.1%	596 (470-687)	26	88.5%	582 (479-774)	21	23.8%	320 (253-390)	17	5.9%	279 (240-301)	17	0.0%	307 (291-350)	12	25.0%	322 (262-396)
D-Dimer DDU (<251 ng/mL)						***MAX***												
ALL	122	98.4%	1585 (924-2191)	93	100.0%	1915 (1295-3145)	82	100.0%	1330 (890-1743)	75	48.0%	245 (175-415)	93	16.1%	155 (135-220)	52	9.6%	135 (135-160)
Mild	41	97.6%	1170 (635-1820)	31	100.0%	1595 (1005-2055)	30	100.0%	1018 (694-1559)	22	50.0%	245 (151-514)	35	11.4%	145 (135-190)	19	5.3%	135 (135-150)
Moderate	43	97.7%	1870 (1095-2438)	35	100.0%	1915 (1358-3220)	32	100.0%	1423 (1039-3259)	34	52.9%	263 (190-423)	33	24.2%	170 (135-240)	21	19.0%	145 (135-195)
Severe	38	100.0%	1743 (1355-2241)	27	100.0%	2330 (1883-4550)	20	100.0%	1475 (985-1800)	19	36.8%	225 (163-303)	25	12.0%	165 (135-210)	12	0.0%	135 (135-138)

Abbreviations: aspartate aminotransferase – AST; alanine transaminase – ALT; white blood cell count – WBC; absolute lymphocyte count – ALC; hemoglobin – Hgb; brain natriuretic peptide – BNP; C-reactive protein – CRP; erythrocyte sedimentation rate – ESR
