# Supplementary figures and images for: Timely Resolution of SARS-CoV-2-Related Multi-System Inflammatory Syndrome in Children
Source: Viruses. 2022 Dec 29;15(1):94. doi: 10.3390/v15010094 (PMC9866445; doi:10.3390/v15010094)

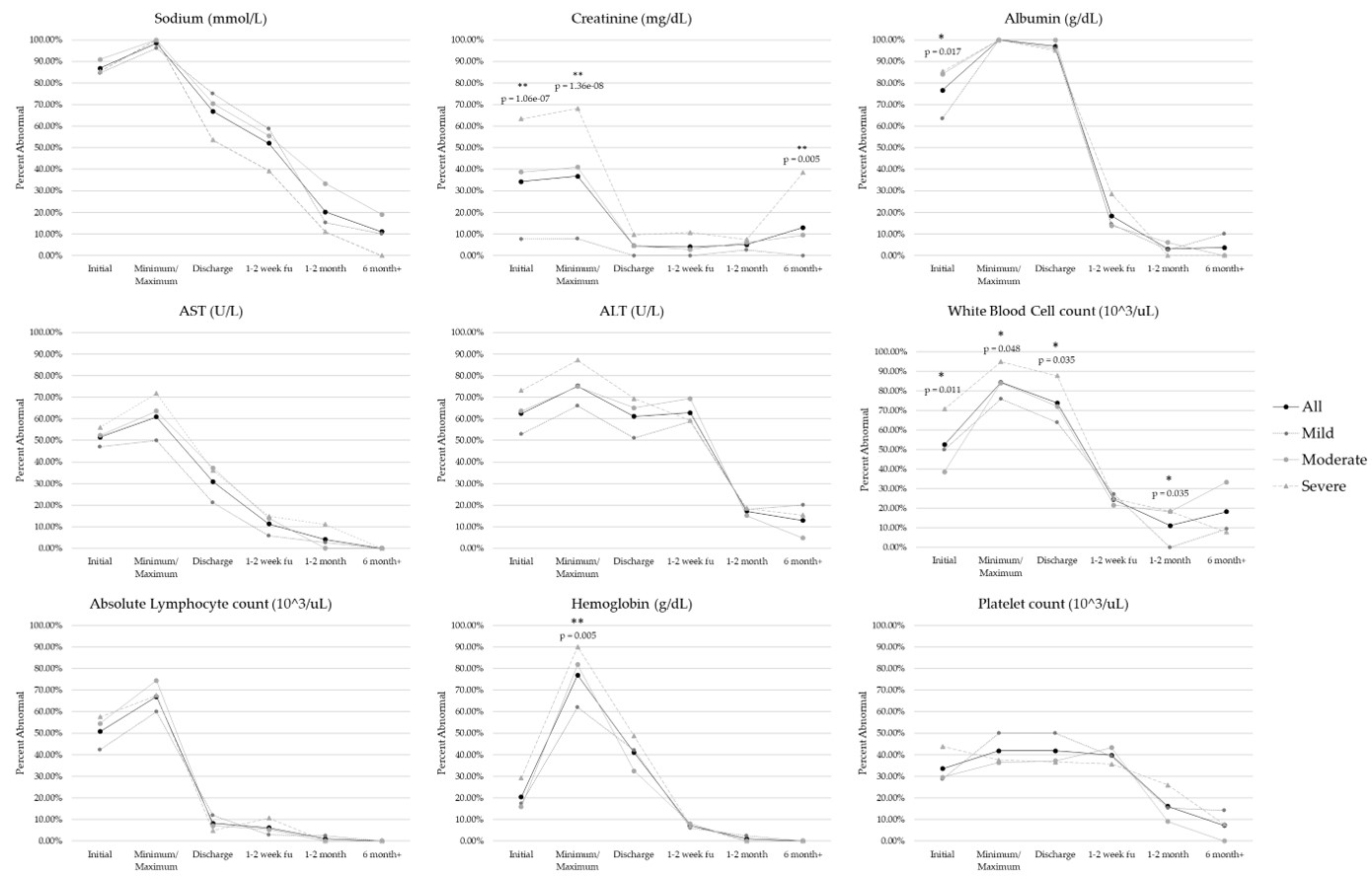

Supplement: Supplementary file 1 [file viruses-15-00094-s001.zip › Supplemental Figure 1.jpg]

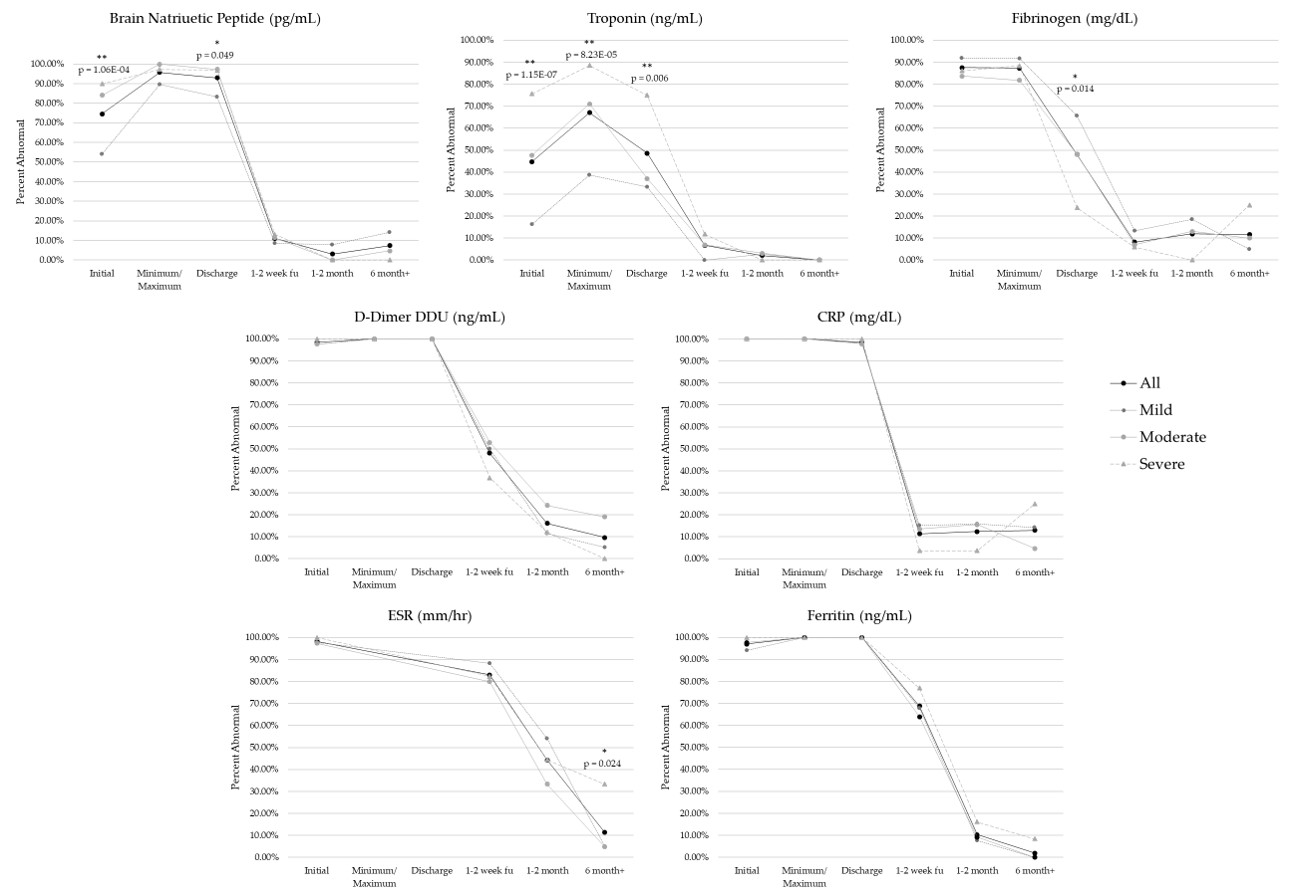

Supplement: Supplementary file 1 [file viruses-15-00094-s001.zip › Supplemental Figure 2.jpg]
